# Evaluation of the Permanence of Land Use Change Induced by Payments for Environmental Services in Quindío, Colombia

**DOI:** 10.1371/journal.pone.0147829

**Published:** 2016-03-01

**Authors:** Stefano Pagiola, Jordi Honey-Rosés, Jaume Freire-González

**Affiliations:** 1 World Bank, Washington D. C., United States of America; 2 School of Community and Regional Planning, University of British Columbia, Vancouver, BC, Canada; 3 ENT Environment and Management, Vilanova i la Geltrú, Spain; University of Waterloo, CANADA

## Abstract

The effectiveness of conservation interventions such as Payments for Environmental Services (PES) is often evaluated—if it is evaluated at all—only at the completion of the intervention. Since gains achieved by the intervention may be lost after it ends, even apparently successful interventions may not result in long-term conservation benefits, a problem known as that of permanence. This paper uses a unique dataset to examine the permanence of land use change induced by a short-term, asset-building PES program implemented in Quindío, Colombia, between 2003 and 2008. This the first PES program to have a control group for comparison. Under this program, PES had been found to have a positive and highly significant impact on land use. To assess the long-term permanence of these changes, both PES recipients and control households were re-surveyed in 2011, four years after the last payment was made. We find that the land use changes that had been induced by PES were broadly sustained in intervening years, with minor differences across specific practices and sub-groups of participants, indicating that these changes were in fact permanent. The patterns of change in the period after the PES program was completed also help better understand the reasons for the program’s success. These results suggest that, at least in the case of productive land uses such as silvopastoral practices under conditions such as those at the study site, asset-building PES programs can be effective at encouraging land owners to adopt environmentally-beneficial land management practices and that the benefits will persist after payments cease.

## Introduction

Payments for environmental services (PES) programs have attracted considerable attention as a strategy to protect natural resources and improve their long-term management [1,2,3,4]. As with many other conservation interventions, there are important questions concerning their effectiveness. One such question concerns the long-term sustainability of their results. However, there have been no empirical analyses to date of this long-term sustainability. The effectiveness of PES program has hitherto been evaluated (if it is evaluated at all), only at the completion of the intervention. Since gains achieved by the intervention may be lost after it ends, even apparently successful interventions may not result in permanent conservation benefits.

PES programs make payments that are conditional on managing natural resources in ways that generate benefits for others [2,3,5,6]. Under the PES approach, the users of environmental services (such as users of clean water) pay those who contribute to generating them (such as upstream land users)—just as they would pay them to supply more tangible services such as maize or milk. Essentially, PES seeks to internalize externalities.

Recent years have seen a substantial growth in the use of PES, particularly in Latin America. PES programs are being implemented in Brazil [7], Colombia [8], Costa Rica [9], Ecuador [10,11], Mexico [12,13], among others, and are under consideration in several other countries. These programs cover a wide range of scales and contexts. National-scale programs are in place in Costa Rica, Ecuador, Mexico, and several Brazilian states. Smaller programs (usually watershed-scale) can be found throughout the continent, in almost every country. Many programs focus on preserving water services, but programs that sequester carbon (for regulated or voluntary markets) are also common, and may become even more common if plans for Reduced Emissions from Deforestation and Degradation (REDD) come to fruition [14,15]. Although few PES programs focus on biodiversity directly, biodiversity conservation is an important secondary objective of many programs.

As with other conservation interventions [16,17], there has been growing concern over the effectiveness of PES [18]. Concerns have been raised that PES may not in fact induce the desired land use changes (that is, that they may lack *additionality*); that any induced land use changes may not in fact generate the desired services (for example, because the wrong land uses were induced, or total land use change was insufficient); that such changes may not be sustainable or *permanent* (because they are abandoned once the program ends); or that second-order impacts of the programs may diminish, or even negate, the benefits of the program (a problem known as *leakage* or *slippage*). There have also been concerns over distributional and social impacts. Despite these concerns, few efforts have been made to assess the impact of PES programs, and most of those have been hampered by lack of data, sometimes leading to very divergent results.

The few evaluations of PES programs that have been conducted have shown mixed results, in part because almost none had a control group to serve as a counterfactual, and few collected baseline data. Costa Rica’s PES program has been the most studied, but the available studies have a wide range of results, ranging from a 10 percent increase in primary forest cover nationwide in 2005 over what it would have been without the PES Program [19]; to a minimal impact on deforestation in 1997–2000 [20], and only slightly higher impacts in 2000–2005 [21]; and an increase in forest cover among PES recipients of about 11–17 percent of the area under contract [22]. In Mexico, Alix-Garcia and others [23], find that the national PES program reduced deforestation among participants by about 50 percent. At a smaller scale, Honey-Rosés and others [24] find that a PES program aimed at conserving the Monarch Butterfly Reserve succeeded in reducing deforestation and forest degradation compared to what it would have been, but not in eliminating it. In Colombia, Pagiola and others [25] find that a PES program achieved substantial and strongly statistically significant land use change among recipients. All these studies focus exclusively on the problem of additionality during implementation. Only Alix-Garcia and others [23] examine the extent of slippage under Mexico’s national PES program, finding that it partially offset the estimated impact on deforestation.

In this paper, we are specifically concerned with the long-term sustainability or permanence of the benefits generated by PES—the least studied aspect of the effectiveness of PES. We use a unique dataset to examine the long-term sustainability of environmentally-beneficial land use change induced by a PES program implemented in Quindío, Colombia, between 2003 and 2008. Under this program, which was the first PES program to have a control group, PES was found to have a positive and highly significant impact on land use, with PES recipients substantially increasing the proportion of farm area devoted to environmentally beneficial land uses [25]. Because of the short-term nature of the program, however, there was considerable concern that gains would be lost once the program ended. To assess the long-term sustainability of these changes, both PES recipients and control households were re-surveyed four years after the last payment was made to measure subsequent changes to land use.

We begin by discussing why evaluating the long-term sustainability of land use change induced by short-term PES programs is important and formulating several hypotheses about long-term outcomes. We then describe the project and its PES mechanism, the Quindío site, and the land uses changes that were induced during implementation of the project. We then use detailed monitoring data collected at the study site to examine the land use changes that took place subsequent to the last payment being made. In particular, we search for any evidence that former PES recipients have abandoned the practices they adopted while receiving payments. We also examine the extent to which some of these land uses may have been further expanded even after payments ended, either by former PES recipients or by control households. We also examine whether different sub-groups of participants behaved differently. It should be stressed that we are not undertaking here an impact evaluation of PES on land use (for which, see [25])/ Rather, we are examining the permanence of PES impacts: whether the gains made under PES are maintained after payments end. We find that the land use changes that had been induced by PES were broadly sustained in intervening years, with minor differences across specific practices and sub-groups of participants. Some practices experienced continued expansion even after the payments ceased. The patterns of change in the period after the PES program was completed also help better understand the reasons for the program’s success. We conclude by discussing the implications of our results for PES program design.

### Long-Term Sustainability of PES Impacts

Most PES programs are designed to be long-term programs, making annual payments to landholders essentially indefinitely (although most PES contracts are typically for five years, the intent is to renew them indefinitely). This is particularly true of PES programs that aim to conserve existing environmentally-beneficial land uses such as forests and prevent their conversion to less desirable land uses. The logic of these programs is that the returns to landholders of environmentally-beneficial land uses are lower than those of alternatives—if this were not case, there would be no pressure to change land use. Accordingly, perpetual payments are necessary to induce landholders to retain such land uses. Payments are made annually, upon verification that landholders have maintained the desired land uses. In such cases, there is no expectation of sustainability once payments end—on the contrary, the expectation is explicitly that the environmentally-beneficial land uses would be abandoned if payments ended. Concerns over sustainability thus focus primarily on the sustainability of the funding sources and the institutional arrangements that allow long-term payments to be made. In such long-term PES programs, the more important concern is that of *additionality*: many participants in such programs may be receiving payments for land uses they would have undertaken anyway, so that the programs generate few or no additional environmental services compared to the no-program counterfactual [18].

Other PES programs, however, only make short-term payments. This is often the case of programs that seek to restore degraded ecosystems, replacing environmentally-harmful land uses with more beneficial ones. Wunder [1] calls programs that focus on restoration “asset-building”, in contrast to “use-restricting” conservation-focused programs. The logic in such cases is that returns to landholders from environmentally-beneficial land uses can exceed those of alternatives once obstacles to their adoption have been overcome. In such cases, a short-term PES program that ‘tips the balance’ between environmentally-harmful and -beneficial land uses may be sufficient. This was the hypothesis of the asset-building PES program examined here.

The hypothesis that short-term payments are sufficient to induce lasting land use change may, however, be mistaken. If returns to environmentally-beneficial land uses are lower than those of alternatives, landholders may still participate in the PES program and temporarily adopt the desired land uses so as to receive the payments (making PES appear to be successful during implementation), but would then abandon these land uses once payments cease. This would, of course, result in the loss of any environmental benefits after the program’s end. The resources used to induce the land use change would thus have been wasted. It is important, therefore, to verify, rather than assume, whether land use changes induced by short-term PES programs are indeed sustained after payments end.

Observing land use changes after completion of a PES program is also important for other reasons. In particular, while some fear that land uses will be abandoned once payments cease, others hope that—on the contrary—PES will lead to widespread adoption even after payments cease. This could occur, for example, if benefits to landholders were not the main obstacles to adoption of environmentally-beneficial land uses. These land uses may not have been adopted prior to the PES program, for example, if landholders were not aware of their benefits, or did not know how to implement them. If the area under the desired land uses continues to expand even after payments end, it may indicate that the apparent benefits of PES may in fact have resulted not from the actual payments but from the technical assistance (TA) that was provided concurrently. In that case, a TA program alone may be sufficient. Alternatively, the primary obstacle to adoption may have been that landholders lacked the financing necessary to undertake the necessary investments. In this case, payments may have affected land use choice by relaxing this constraint rather than through their effect on profitability. In the latter case, a credit program may be an alternative to PES.

The permanence of PES-induced land use changes may differ across land uses, as the relative profitability and technical complexity of environmentally-friendly land uses vary. Thus, some land uses may prove to be sustainably adopted thanks to an asset-building PES programs while others are not. Impacts may also vary across participants. Poorer households, for example, may have greater financing constraints than better-off households. These differences, if observed, would have important implications for PES program design. Any evaluation should thus seek to identify such differences in impacts rather than looking at average impacts.

To date, the only effort to assess the long-term permanence of a PES program has been a study of China’s Sloping Land Conversion Programs (SLCP) [26]. This study, however, used stated preference techniques to try to predict whether the program’s effects would prove permanent, rather than observations of actual post-program behavior.

## Methods

This paper uses data from an asset-building PES program implemented from 2003 to 2007 to examine whether land use changes induced by PES are maintained once payments end. The *Regional Integrated Silvopastoral Ecosystem Management Project* (hereafter the ‘Silvopastoral Project’) used PES to encourage landholders to adopt silvopastoral practices on degraded and treeless pastures, so as to generate increased biodiversity conservation and carbon sequestration [27]. To examine the long-term sustainability of these land uses changes, we re-surveyed all former participants four years after the last payment was made. The Silvopastoral Project was implemented at three pilot sites: Quindío, in Colombia; Esparza, in Costa Rica; and Matiguás-Río Blanco, in Nicaragua. Here we use data from the Quindío site.

This paper is based on data collected beginning in 2002. At that time, we did not have a review board, and we still do not (we often work with universities, and so rely on their review boards, but this was not the case in this project). All participants in the project (and survey) volunteered to participate. Open meetings were held to explain the project and its requirements (including the fact that there would be intensive monitoring), and anyone interested was invited to fill an application and return it, thus providing written, informed consent to participate. No personally identifiable information is provided on any of the participants.

### The Silvopastoral Project

The Silvopastoral Project piloted the use of PES to encourage the adoption of silvopastoral practices [27]. It was developed with support of the multi-donor Livestock, Environment and Development Initiative (LEAD), hosted by the Food and Agriculture Organisation (FAO). The project was implemented by the World Bank and financed by a US$4.5 million grant from the Global Environment Facility (GEF). Local non-governmental organizations (NGOs) implemented the project in the field. The Centre for Research on Sustainable Agricultural Production Systems (*Centro para la Investigación en Sistemas Sostenibles de Producción Agropecuaria*, CIPAV) was responsible for implementing the project in Quindío.

The expansion of cattle production has been an important factor in the degradation of natural habitat and biodiversity in Latin America [28,29,30]. Traditional livestock production practices based on extensive grazing have also often proven unsustainable, with soil fertility being depleted and vegetation cover diminishing after an initial period of high yields, resulting in soil erosion, water supply contamination, air pollution, further biodiversity loss, and landscape degradation. Falling producer income can result in continuing poverty and lead to pressure to clear additional areas.

Silvopastoral practices introduce trees in livestock production systems. They include (1) planting trees and shrubs within pastures; (2) establishing cut and carry systems, which use the foliage of trees and shrubs planted in ‘fodder banks’ as livestock feed; and (3) using fast-growing trees and shrubs for fencing and wind screens. Such practices provide deeply rooting, perennial vegetation that is persistently growing and has a dense but uneven canopy.

Landholders derive a variety of benefits from silvopastoral practices, including using the products of the tree component (such as fruit, fuelwood, fodder, or timber); maintaining or improving pasture productivity by increasing nutrient recycling; and diversifying production [31]. However, silvopastoral practices also offer numerous other benefits that do not benefit landholders directly. Silvopastoral practices have important biodiversity benefits, because of their increased complexity relative to traditional pastures—they can: provide scarce resources and refuge for wildlife species, thus helping their survival; help propagate native forest plants; provide shelter for wild birds; and help connect protected areas [32,33,34]. Silvopastoral practices also help mitigate climate change by fixing significant amounts of carbon in the soil and in the standing tree biomass [35,36]. They can also affect water services by promoting infiltration and slowing runoff and erosion, though the specific impact is likely to be site specific [30,37].

As biodiversity conservation, carbon sequestration, and watershed protection benefits are experienced off-site, landholders will not normally take them into account when deciding which practices to adopt. However, the on-site benefits alone may be insufficient by themselves to induce landholders to adopt silvopastoral practices—particularly practices with substantial tree components, which have high initial planting costs and may not bring benefits until several years later. Estimates prepared for the project show rates of return to landholders of adopting silvopastoral practices of between 4 and 14 percent [38]. Similarly, White and others [39], found rates of return of 9 to 12 percent to adopting improved pasture in Esparza, Costa Rica. As a result of these low rates of return, adoption of silvopastoral practices is often low.

The Silvopastoral Project sought to improve the adoption of silvopastoral practices by offering payments proportional to their expected biodiversity and carbon sequestration benefits (watershed benefits were not considered in this project). To do so, the project developed an ‘environmental services index’ (ESI) which aggregated indices of the biodiversity conservation and carbon sequestration services associated with specific silvopastoral practices [40]. The biodiversity conservation index was scaled with the most biodiversity-poor practice (annual crops) set at 0.0 and the most biodiversity-rich practice (primary forest) set at 1.0. A panel of experts assigned points to each specific land use within this spectrum, taking into consideration factors such as the number of and type of species, their spatial arrangement, and stratification. A similar procedure was used to establish the carbon sequestration index, with different land uses given points according to their capacity to sequester stable carbon in the soil and in biomass. The project distinguished 28 different practices, each with its own ESI score (see Table 1). Annual payments were based on the change in the total ESI score for the entire farm compared to its ESI score at the beginning of the project, with each incremental ESI point being worth US$75 per year, over a four-year period. Baseline ESI points received an initial, one-time payment of US$10/point.

**Table 1:** Land use at the Silvopastoral Project site, Quindío, Colombia. (% of farm, unless otherwise indicated).

		*PES recipients*			*Control group*		
*Land use*	*Environmental services index (points/ha)*	*2003*	*2007*	*2011*	*2003*	*2007*	*2011*
Annual crops	0.0	1.3	1.3	3.0	7.7	11.3	8.1
Degraded pasture	0.0	2.8	0.3	0.7	1.9	1.0	1.4
Natural pasture without trees	0.2	24.8	8.1	9.9	6.8	3.8	2.2
Improved pasture without trees	0.5	37.3	30.4	23.4	51.2	42.7	30.7
Semi-permanent crops (plantain, sun coffee)	0.5	6.5	5.1	5.2	13.6	15.4	24.0
Natural pasture with low tree density (< 30/ha)	0.6	0.2	0.4	2.0	0.0	1.4	1.8
Diversified fruit crops	0.7	2.5	1.9	3.0	0.3	1.8	4.2
Fodder banks^a^	0.8	0.2	1.0	1.0	0.0	0.5	0.8
Improved pasture with low tree density (< 30/ha)	0.9	1.9	11.4	9.6	0.8	1.6	5.3
Natural pasture with high tree density (>30/ha)^b^	1.0	0.0	2.3	2.7	0.0	0.0	0.0
Shade-grown coffee	1.3	0.8	1.3	0.9	1.0	1.0	1.3
Improved pasture with high tree density (>30/ha)^b^	1.3	0.1	9.3	10.0	0.0	0.0	0.1
Bamboo (guadua) forest	1.3	1.5	1.8	1.8	0.2	0.2	0.2
Timber plantation^a^	1.4	0.0	0.2	0.3	0.3	0.3	0.3
Riparian forest	1.5	12.9	13.7	14.0	10.4	10.4	10.6
Intensive silvopastoral system (iSPS)	1.6	0.0	4.4	4.8	0.0	2.9	3.0
Primary and secondary forest^a^	1.9	7.3	7.1	7.7	5.9	5.8	6.1
**Total area**		**100.0**	**100.0**	**100.0**	**100.0**	**100.0**	**100.0**
Multistory live fence or wind break (km)^b^	1.1	2.1	356.9	386.4	3.0	13.6	16.6

*Notes*: Totals may not add up because of rounding.

a. Similar land uses with small areas have been aggregated.

b. The project distinguishes land uses with recently planted trees from the same land uses with mature trees for the purpose of computing the ESI score; here these land uses have been aggregated to their mature state and the corresponding ESI score is shown.

*Source*: ESI from CIPAV (2004); area from Silvopastoral Project mapping data.

The Silvopastoral Project made payments for baseline ESI points in July 2003. After monitoring land use changes, it then made payments for incremental ESI points (measured relative to baseline points) each year from 2004 to 2007. Since 2007, the former program participants have received no systematic support, in terms of either payments or TA, from CIPAV. However, some have received occasional visits from CIPAV.

### Study Site

**Fig 1:**
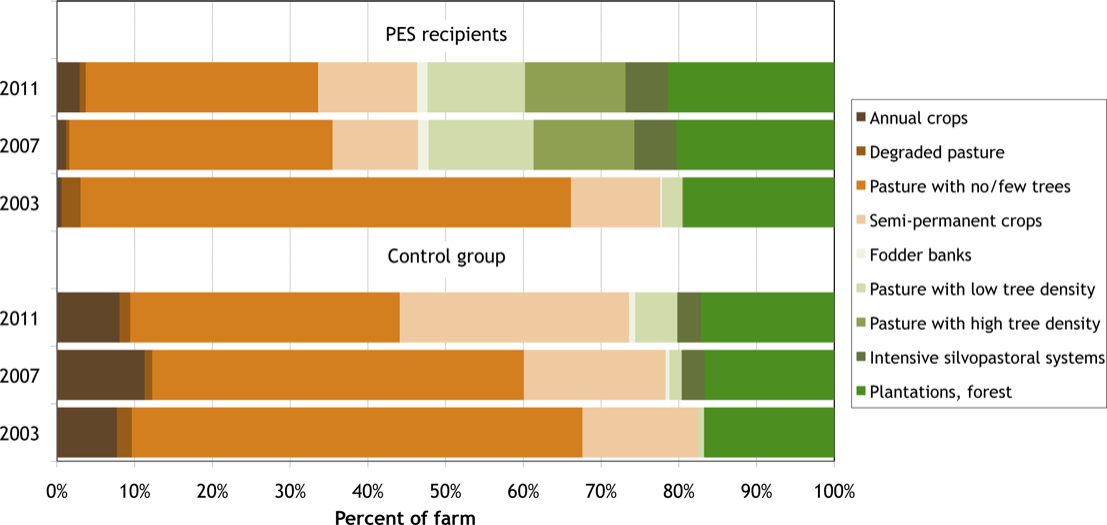
Observed land use in Quindío, Colombia, 2003–2011.

The Quindío area is located in the watershed of Río La Vieja, in Colombia’s Central Cordillera, at an altitude of about 900–1,300m above sea level. Farms range in size from 10-20ha to 50-80ha. Larger farms are usually owned by urban professionals and managed by employees (*mayordomos*). Farm households range from extremely poor to quite wealthy. At project start, extensive grazing was the main land use in Quindío, with degraded and treeless pastures accounting for about 65 percent of the area (Table 1 and Fig 1). Livestock production was primarily for meat production, with a small proportion being used for milk production. Other than some forest remnants—primarily riparian forest—overall tree cover was low. Some farms—particularly lower-income farms—had small areas dedicated to other productive activities, such as semi-permanent crops (mostly bananas), fruit crops, shade-grown coffee, and annual crops. There were practically no silvopastoral practices such as pastures with trees, fodder banks, or live fences. For example, only 7 in 110 farms surveyed had fodder banks at project start, with an average of less than 1ha each.

### Treatment Group

Budget constraints limited the number of participants in Quindío to 80 households. To enroll them, public workshops were held to explain the project, with the support of the Quindío livestock association, and field visits were organized to an area where silvopastoral practices were already in use. Households who expressed an interest and met minimal criteria on herd size were then accepted on a first-come basis.

The two primary treatments of interest were payments and technical assistance (TA). All households in the treatment group were offered PES, but 56 households were randomly selected among them to also receive on-farm TA. Although all participants received advice on which land uses might be most appropriate on their farms, the TA sub-group also received on-farm, in-person guidance on how to implement the selected land uses. The design made it possible to compare the effect of PES alone and the combination of PES and TA to the control group, which received neither. The treatment group was then further sub-divided, with half receiving payments for all four years of the project, while the other half only received payments for two years. The intent was to allow an early assessment of whether land use change would prove sustainable once payments ended. Payment levels were slightly higher for the 2-year group, to compensate for the shorter duration of the payments, so that in principle the payment received to adopt a given land use should have been roughly similar in present value terms for members of both groups. Households were randomly assigned to either the 4-year or the 2-year group. There were thus effectively four treatments: PES for either 2 or 4 years, both either with intensive TA or without it. Among PES recipients, there were no significant differences in household characteristics among the sub-groups. Table 2 shows the characteristics of PES recipient households.

**Table 2:** Characteristics of participating households, Quindío, Colombia.

*Variable*	*PES only*	*PES and TA*	*PES all*	*Control group*	*Entire sample*
Income per capita (million COP)	5.1	9.5	8.0	14.2	9.8
Assets (million COP)	9.4	8.3	8.7	8.7	8.7
Farm area (ha)	25.8	31.5	29.6	25.4	28.3
Cattle (livestock units)	59.7	49.9	53.3	48.5	51.9
Flat (% farm area)	26.2	20.4	22.4	36.9	26.7
Distance to nearest village (km)	6.7	6.9	6.8	5.2	6.4
Water (% with water service)	95.8	93.3	94.2	96.6	94.9
Farm resident (%)	33.3	31.1	31.9	17.2	27.6
Family labor (man-days/ha/yr)	7.2	9.5	8.7	nd	nd
Household size (members)	5.1	4.6	4.8^a^	3.7^a^	4.5
Dependency ratio (children per adult)	0.4	0.4	0.4^a^	0.2^a^	0.4
Age of household head (years)	45.2	42.6	43.5	43.9	43.6
Literacy of household head (%)	100.0	93.3	95.7	93.1	94.9
Education of household head (years)	5.2	5.0	5.1	4.3	4.9
Off-farm work (% with off-farm employment)	12.5	15.6	14.5	10.3	13.3
Technical assistance (% with current access)	45.8	31.1	36.2^a^	10.3^a^	28.6
Credit (% with access to credit)	20.8	31.1	27.5	13.8	23.5
Number of observations	24	45	69	29	98

Notes: Data reflects conditions just prior to project start.

^a^ indicate means are significantly different in paired t-test at 10% test level. nd = no data.

Children are household members under 12.

Livestock are converted into livestock units (*Unidad Gran Ganado*, UGG) using the following conversion factors: adult cows, 1.0 UGG; oxen or breeding bulls, 1.55 UGG; calves, 0.33 UGG; yearlings, 0.7 UGG.

*Source*: Silvopastoral Project baseline survey.

### Control Group

The Silvopastoral Project included a control group to allow project-induced land use changes to be distinguished from changes induced by other factors. In fact, it was the first PES project anywhere to include a control group. Ideally, applicants would have been randomly assigned to either the treatment or the control group [16,41]. This was not feasible, however, as the treatment group had already been selected when the decision to include a control group was made. Fortunately, the number of applications received was sufficient that a control group could be selected from among applicants who had been unable to access the program. As applications had been accepted on a first come, first served basis, there was no reason to expect that rejected applicants differed systematically from accepted applicants. Indeed, control households have similar characteristics (in terms of size, land use patters, and agro-ecological conditions) to PES recipient households (Table 2). Budget constraints limited the control group to 30 households.

### Data Collection

Baseline data on household characteristics of all PES recipients and control households at the site was collected through a survey conducted in late 2002, during project preparation. All former PES recipients and control households were then re-surveyed in mid-2011, four year after the PES program ended. The questionnaire for the new survey was based on that of the 2002 baseline survey, but also included questions on the motivations for maintaining, extending, or reducing the use of different land uses in the period since the end of the project. The baseline survey covered 110 households, but nine observations were later dropped, either because households sold their land and moved away, or because the household head died. Two additional households were dropped on common support grounds (their area exceeded 200ha, while all control group farms were smaller than 200ha). The final data set thus covered 99 households: 70 PES recipients and 29 control group members.

Land use changes were tracked using detailed land use maps prepared annually from 2002 to 2007 for each farm in the PES recipient and control groups, based on remote sensing imagery (Quickbird imagery with 0.6m resolution) and field visits. Each land use in each plot was matched to one of the ESI’s 28 land uses, providing accurate and consistent measures of area and ensuring that land uses are classified consistently. At the same time as the 2011 survey, the land use maps for each participant were updated, using the same methodology as was used during the Silvopastoral Project (by some of the same personnel, or by new personnel that had been trained by Silvopastoral Project personnel) to ensure consistency with the previous land use maps.

### Outcome Measurement

The Silvopastoral Project differed from most PES programs (and from many other development programs aimed at landholders) by offering a large menu of land use options that participants could choose from, in light of their own preferences and constraints, rather than focusing on a small number of preferred land uses. As such, the outcome cannot be expressed by a binary participation/non-participation variable. An outcome variable is needed that captures the extent and nature of land use changes by PES recipient and control households. The *area converted* gives a first cut, but is constrained by farm size. Households with smaller farms may appear to be participating less simply because they have less land. The *proportion of farm area converted* has the opposite problem: smaller farms may appear to participating ‘more’ than larger ones even though they are converting smaller areas, and so having a smaller environmental impact. Any indicator based on hectares also fails to capture the quality of changes. Sowing improved grasses in a treeless pasture generates lower environmental benefits than converting it to pasture with high tree density, but will appear the same have in terms of either area converted or percent of farm area converted. Investments such as establishing live fences are also difficult to incorporate into area-based indicators. An alternative approach is to weight the area converted by the ESI of the land use change, and then add the points for live fencing. This measure can also be stated in different ways. The simplest measure is the *increase in total ESI*, but like area converted is constrained by total farm size. The *increase in ESI per hectare* and the *percent increase in ESI* avoid these problems. As each of these alternatives captures different aspects of the problem, we use them all at different points.

## Results

We begin by briefly reviewing the results of the Silvopastoral Project during its implementation period; these results are examined in more detail by Pagiola and Rios [25]. We then examine how these results changed in the three years after the project ended.

### Participating Households

Table 2 summarizes the characteristics of participating households (which only includes households still active at the end of the project). The average participating household had 4.5 members, and about 36ha of land, and a herd of about 57 livestock units. They had an average per capita income of about COP10 million. As can be seen in Table 2, PES recipient households had slightly different average characteristics from control group households, but most differences were not significant. This is to be expected, given the relatively small sample size and the diversity of conditions in the area.

### Changes in Land Use Induced by PES

**Fig 2:**
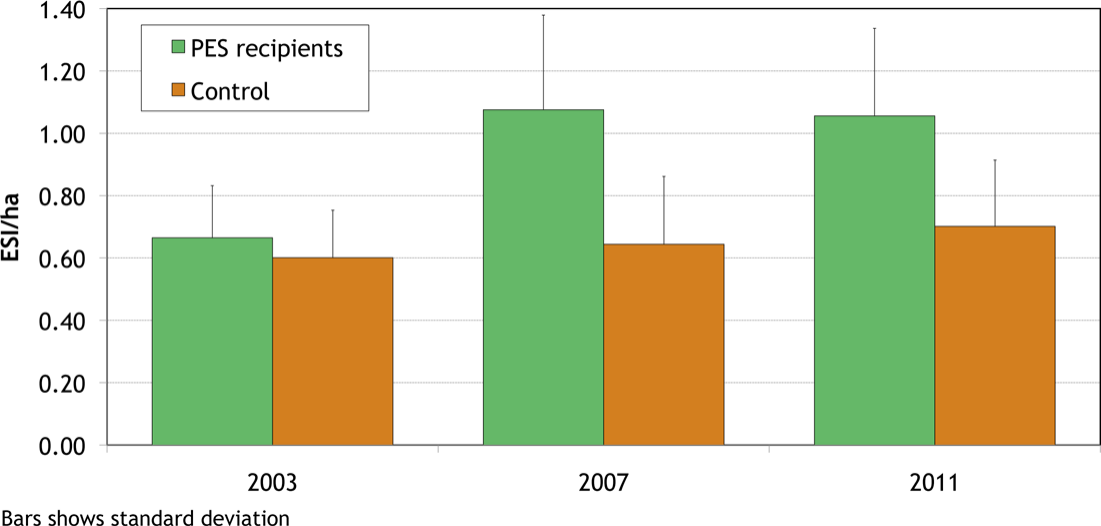
Environmental service generation in Quindío, Colombia, 2003–2011.

Table 1 and Fig 1 compare land use by PES recipients in 2003 (the project’s start), 2007 (the project’s end), and 2011 (four years after the project’s end). During its implementation, the PES program induced substantial land use change: almost 44 percent of the farm area of PES recipient households experienced some form of land use change between 2003 and 2007. Some observed changes were minor (such as sowing improved grasses in degraded pastures), while others were substantial (such as planting high-density tree stands or establishing fodder banks). Substantial reductions were observed in the area of degraded pasture (which fell by over 90 percent of its original area) and of treeless pasture (which fell by two thirds of its original area). (Note that these figures are for *net* changes in the area under each practice and so understate total changes. For example, while some treeless pastures were converted to natural pastures with high tree density, some natural pastures that already had high tree density were converted to improved pastures with high tree density, reducing the apparent net increase in natural pastures with high tree density.) The area under annual and semi-permanent crops also declined. The greatest increase, of 334ha, was observed in pastures with high tree density. Other practices that were widely adopted include intensive silvopastoral systems (iSPS: *Leucaena* planted at 5,000 trees/ha), whose areas went from nothing to 130ha, and fodder banks, whose area rose from less than 5ha to over 28ha. About 346km of live fencing were established. However, timber plantations and pure forest uses found little favor, with their total area increasing by only 29ha. Overall, these changes resulted in the ESI/ha of PES recipients increasing by over 60 percent (significant at 1 percent), as shown in Fig 2.

In contrast, control households undertook substantially fewer land use changes in the same time period (Table 1 and Fig 1). Control households converted less than 13 percent of their land area, and adopted substantially less beneficial land uses, for an increase in ESI/ha of only 7 percent (which is not statistically significant). Econometric and difference-in-difference analyses confirm that these differences are statistically significant and did not result from differences in household characteristics between PES recipient and control group households [24]. These results conclusively demonstrate that the land use changes induced by PES in Quindío were *additional*.

Among the possible land use changes, Pagiola and Rios [24] found that there was very little adoption of more conservation-oriented land uses (such as secondary forests), even as production-oriented land uses (such as fodder banks) were adopted extensively—even though the payments offered by the project for conservation-oriented land uses were higher. Pagiola and others [41] also found that the extent and nature of changes made by relatively poorer households were not significantly different from those of relatively better off households. More surprisingly, Pagiola and Rios [42] found that TA recipients did not undertake significantly more or better land use changes.

### Post-PES Land Use Changes

As noted, there was concern that the environmental gains made during implementation of the PES program would be temporary, with previous land uses returning once payments ended. The inclusion of a sub-group that would receive payments only over two years was an initial effort to determine whether these concerns were well founded. Pagiola and Rios [24] found no significant differences between 2-year and 4-year PES recipients in land use change, at the time of the project’s end. This result was promising, but did not entirely allay the concerns, as the continued presence of monitoring teams during the remaining two years could have inhibited 2-year PES recipients from abandoning the land uses they had adopted.

**Fig 3:**
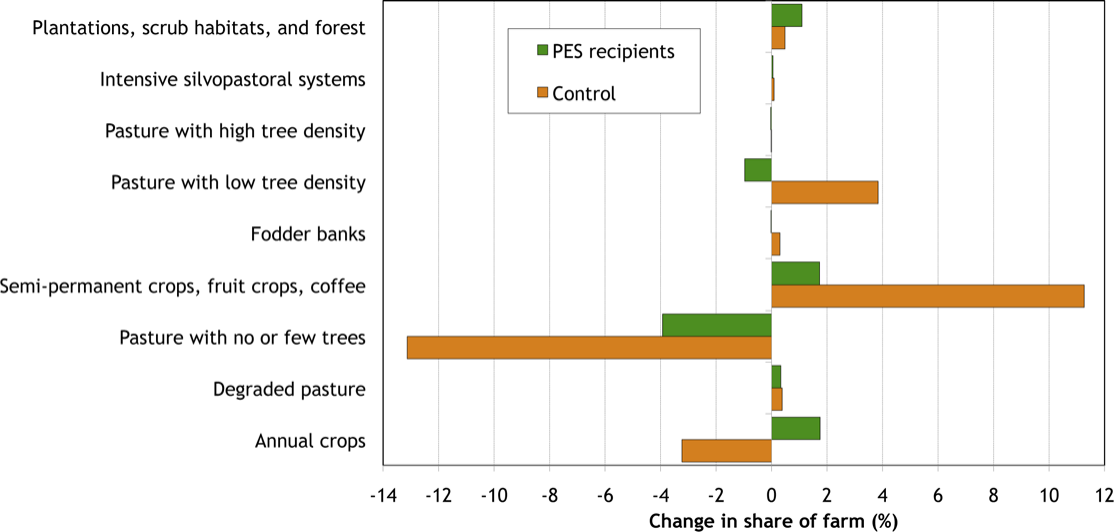
Observed changes in land use in post-PES period in Quindío, Colombia, 2007 to 2011.

Table 1 and Fig 1 show the observed land use changes in the four years since the PES programs ended, and Fig 3 examines the observed post-PES changes in more detail. Among former PES recipients, these changes were minimal. The main change was a continued decline in the area of treeless pasture, which fell by about 4 percent of their farm area (compared to a fall of almost 24 percent of farm area during the project). Some of this area, however, was converted to annual crops and so did not bring any additional environmental benefits. Among environmentally-beneficial land uses, there was some very minor expansion (about 1 percent of farm area) of secondary and riparian forests and of semi-permanent crops—specifically, monoculture fruit tree plantations. Among silvopastoral practices there were very few changes, except for a small decline (less than 1 percent of farm area) of pasture with low tree density.

Land use changes in the post-project period among former control households were somewhat larger in terms of area, but very limited in terms of their extent, with a significant fall (over 13 percent of their farm area) in the area under degraded pasture, most of which was converted to semi-permanent crops—primarily un-shaded perennials (Fig 3). Indeed, observed changes among former control households are driven by a small number of farms converting areas of degraded pasture to semi-permanent crops––a land use change the Silvopastoral Project had not emphasized as it brings very limited environmental improvements. There was very limited adoption of any silvopastoral practice, with the sole exception of pastures with low tree density (adopted on less than 4 percent of their farm area).

As a result of these changes, the overall ESI/ha of former PES recipients declined slightly (by less than 2 percent), and that of control households increased (by almost 9 percent) but neither change was statistically significant (Fig 2).

**Fig 4:**
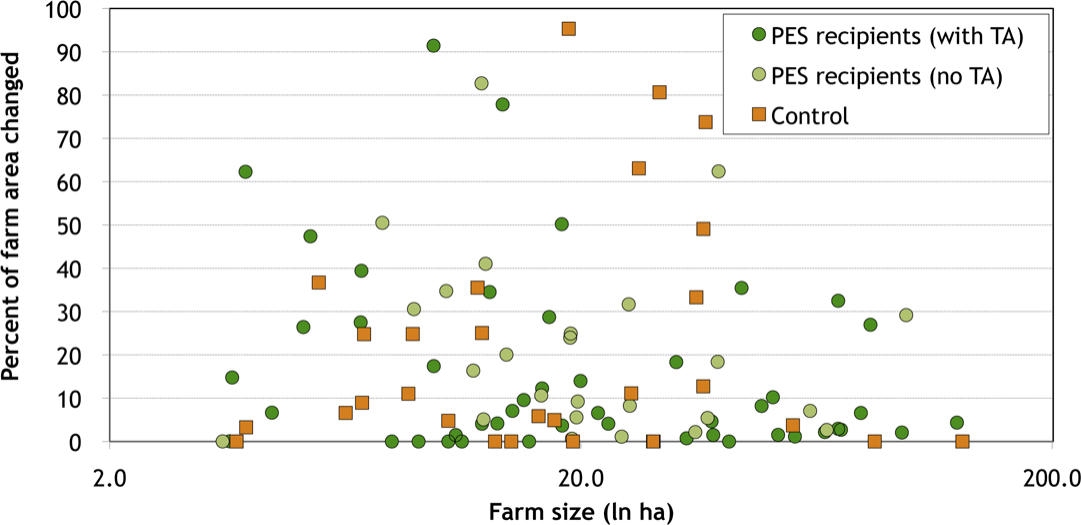
Post-PES land use changes in Quindío, Colombia, by farm size.

**Fig 5:**
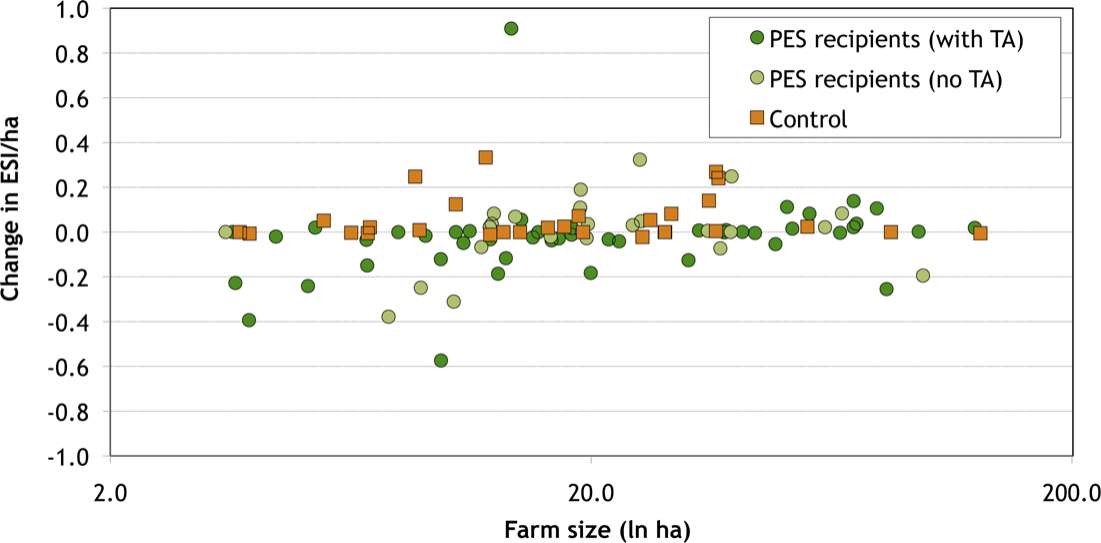
Post-PES changes in ESI/ha in Quindío, Colombia, by farm size.

The observed changes are concentrated among a small group of farmers: 56 percent of PES recipients and 48 percent of control households changed less than 10 percent of their farm area, while 9 percent of PES recipients and 14 percent of control households changed more than half of their farm area. The households which made substantial changes appear to have little in common, however. As can be seen in Fig 4, for example, there is no obvious relationship between farm size and the extent of post-PES land use changes (in terms of proportion of farm area converted), among either PES recipients or control households; being a TA recipient also does not appear to have made a difference. Likewise, as can be seen in Fig 5, there is no correlation between farm size and whether the changes made are environmentally beneficial (as measured by changes in the ESI). Both the positive and the negative outliers in terms of environmental impacts of post-PES changes, for example, are former PES recipients who had received TA. Econometric analysis confirms that having been a PES recipient had no statistically significant impact on post-project changes (see Table B in S1 File). Income levels are likewise non-significant.

The evolution of intensive silvopastoral systems (iSPS) illustrates some of the observed changes in more detail. iSPS is of particular interest, as this practice is considered particularly promising in terms of environmental and economic benefits. While costly and technically complex to implement, iSPS can raise carrying capacity from about half a head of cattle per hectare under extensive grazing to as much as five head per hectare. Prior to the project, the practice had been completely unknown in Quindío. During the course of the project, iSPS were adopted by 26 PES recipient households (a third of the total) on 130ha, with areas ranging from 0.1ha to over 43ha, representing between nearly 0 and over 55 percent of their farm areas, as well as by one control household (on 21.5ha, or 75 percent of its farm). iSPS adopters included some of the smallest farms and some of the largest. Non-adopters were also found throughout the size range. The largest areas of iSPS were found on larger farms, but smaller farms adopted iSPS on relatively larger shares of their farms. Both poor and well-off households were represented among iSPS adopters. Poorer households adopted iSPS on smaller areas, but differences in the portion of farm area dedicated to iSPS were not statistically significant. These results suggested that iSPS could be profitable even without PES. Based on these results, a follow-up project at first did not offer any payments for iSPS adoption, only credit and TA. In the four years following the end of the project, the overall area under iSPS increased by over 7 percent, seemingly confirming this result. This average, however, masks considerable variation: Of previous adopters, 5 households did not change their area under iSPS, 9 households abandoned iSPS entirely, 9 households reduced the area under iSPS (with the biggest reduction being less than 3ha), and 4 households increased the area under iSPS (by an average of 7ha). Moreover, 3 previous PES recipients and one control household that had not adopted iSPS under the project did so after the project’s end, on an average of 1.5ha.

## Discussion

PES recipients undertook substantial land use changes in the years in which they were receiving payments, far exceeding the changes undertaken by control households in terms of both quantity and quality. In an equivalent period following the end of the project, however, they undertook only minor land use changes. These results show that beneficial land uses adopted under the PES program were retained even after payments ceased. At the same time, we do not see evidence that adoption of silvopastoral practices continued spontaneously on any significant scale even in the absence of payments, as had been hoped. This implies that economic incentives, rather awareness or know-how, were the main barrier to adoption.

The widespread adoption of silvopastoral practices during the project indicates that they were more profitable at the study site than alternative land uses when supported by PES. Conversely, that their area did not expand after payments ended indicates that they were not more profitable than alternative land uses without PES. At the same time, their permanence after payments ceased strongly suggests that *once they are established* they remain more profitable than alternative land uses even without PES. Had that not been the case, it would have been simple for landholders to remove them, and they would have suffered no penalties from doing so. These results thus support the hypothesis that financial profitability of silvopastoral practices was the main obstacle to their adoption: that is, that by reducing the initial costs of adoption and providing some income in the period before silvopastoral practices begin to generate sufficient benefits to be profitable, the payments ‘tipped the balance’ towards adoption.

Other possible explanations for the lack of adoption of silvopastoral practices are inconsistent with the observed results. Simple ignorance of their possible benefits, or of how to implement them, were plausible explanations for lack of adoption prior to the project start, when such practices were practically non-existent in the landscape. After four years in which the use of silvopastoral practices expanded dramatically in the Quindío area, these explanations are no longer plausible. If these had been the main obstacles to the adoption of silvopastoral practices, the area under these practices would have continued to expand even in the absence of payments, and particularly so among landholders who received TA. Yet there was very limited expansion, and no significant differences in the extent of such expansion between those who received TA under the project and those that did not. Likewise, if the primary constraint had been the inability to finance the required investments, expansion should have continued even without payments at least among better-off households, and perhaps even among poorer households, as the higher income generated by previously-adopted silvopastoral practices could have financed additional adoption.

The observed changes in land use among former control households also support these conclusions. Lack of knowledge about silvopastoral practices can no longer be blamed, as by 2011 control households had ample time to observe such practices; indeed, some of them had adopted silvopastoral practices themselves. Lack of experience is superficially more plausible as an explanation, as these households never received TA nor even basic guidance on selecting appropriate practices. But, again, such inexperience did not prove absolute obstacles as several control households have implemented silvopastoral practices—including, in two cases, the most complex practices on offer (iSPS). The lack of adoption among control group households is also inconsistent with the hypothesis that former PES recipients did not continue expanding silvopastoral practices because they had no scope to do so, having achieved their optimal land use mix during the project. Control group households, who had only adopted silvopastoral practices to a very limited extent during the project, would have had plenty of scope to adopt these practices in the following four years if they were in fact profitable without PES.

## Conclusions

The Silvopastoral Project was the first PES program to have a control group that was monitored from before the treatment began, which allowed strong conclusions to be reached concerning its effectiveness. It is also the only such program in which additional data was collected on results several years after the project ended, allowing the permanence of its results to be assessed.

The experience of the Silvopastoral Project in Quindío indicates that the PES program has resulted in additional positive land use changes in terms of both the area affected and the nature of the changes. Our results show that concerns about non-permanence of land use changes were unfounded: land uses adopted under the PES program were not abandoned once payments ended.

In addition to the obvious dangers of generalizing from a single result, it is important to note the limitations of these conclusions. First, it should be emphasized that the conclusions apply to an “asset-building” PES program, in which payments are targeted primarily at productive activities (which also generate environmental benefits) rather than at pure conservation activities. These results should not create any expectation that “use-restricting” PES programs aimed at conserving existing environmentally-beneficial land uses could be sustainable without payments. In fact, if the land uses supported by such a use-restricting program were maintained after payments cease, it would likely indicate that the program was non-additional. Second, even among “asset-building” programs, the Silvopastoral Project was unusual in offering a very broad menu of options. Farmers were thus able to select the land uses that were best suited to their conditions, and were thus are less likely to find them a poor fit once payments end.

In addition to showing that PES-induced land use changes were sustainable, these results are also useful in that they help improve our understanding of the reasons why the original project was successful. That environmentally-beneficial land uses expanded rapidly when payments were offered for their adoption but then remained essentially unchanged once payments ended is consistent with the hypothesis that limited profitability was the primary obstacle to their adoption, and inconsistent with several other plausible hypotheses, including that the primary obstacles were lack of knowledge of these practices or of how to implement them, or lack of financing for the required investments. Again, this is not to say that these alternative hypotheses may not be correct in other cases. Indeed, we make no claim to external validity. The very characteristics that make us confident that we have a valid control group may well imply that our participants may be unrepresentative of the farm population as a whole, as all were drawn from the same group of more informed, better connected ‘early adopters’.

A follow-up project, the *Mainstreaming Sustainable Cattle Ranching Project*, is now being implemented in Colombia with the support of the World Bank and funding from the GEF and the United Kingdom’s Department of Energy and Climate Change (DECC). This project, which promotes similar land use changes and uses a similar payment mechanism, is being implemented at five sites across the country, under a range of agro-ecological and socio-economic conditions. It, too, will be the subject of an impact evaluation, thus further improve our understanding of the effectiveness of PES, including its long-term sustainability.

## Supporting Information

S1 File Regression analysis of post-PES changes. (DOCX)
