# Supplementary material for: Evaluation of the Permanence of Land Use Change Induced by Payments for Environmental Services in Quindío, Colombia
Source: PLoS One. 2016 Mar 1;11(3):e0147829. doi: 10.1371/journal.pone.0147829 (PMC4773100; doi:10.1371/journal.pone.0147829)
Supplement: S1 File — (DOCX) [file pone.0147829.s001.docx]

**S1 Supporting Information**

**Evaluation of the Permanence of Land Use Change Induced by**

**Payments for Environmental Services in Quindío, Colombia**

**Supplementary materials: Regression analysis of post-PES changes**

Stefano Pagiola, Jordi Honey-Rosés, and Jaume Freire-González

The analysis of the results of the Silvopastoral Project from 2003 to 2007 showed that some farm characteristics affected the extent to which environmentally-beneficial land uses were adopted (Pagiola and Rios, 2013).

In examining the permanence of these land use changes following the end of payments, we were similarly interested in determining whether there were any differences among sub-groups of participants. Should permanence be particularly low among some particular sub-group, for example, future PES programs might either avoid enrolling farmers from that sub-group or take additional measures to improve the degree to which they maintain land use changes (which might require additional research to better understand the reasons for their lack of permanence).

As described in the main paper, post-PES land use changes were limited in degree among both former PES recipients and former control group members (see Figures 4 and 5 in the main text). Within both groups, a few individual households accounted for the bulk of observed changes. There is no apparent pattern in which households carried out change post-PES. For example, there is no obvious relationship between farm size and the extent of post-PES land use changes (in terms of proportion of farm area converted), among either PES recipients or control households; having been a TA recipient also does not appear affect post-PES changes. Likewise, there is no apparent correlation between farm size and whether the changes made are environmentally beneficial (as measured by changes in the ESI). Both the positive and the negative outliers in terms of environmental impacts of post-PES changes, for example, are former PES recipients who had received TA.

As simple plots such as Figures 4 and 5 can hide underlying relationships, we undertook a regression analysis in order to better ascertain whether farm or household characteristics affected post-PES land use changes. This allowed us to control for other factors that may explain the maintenance of silvopastoral practices during this time period.

We focus on changes in ESI scores per hectare as this indicator captures both area changed and the extent to which the changes made were environmentally beneficial (or harmful).

We included three groups of variables in the specifications: land variables (area and land sold), socioeconomic variables (gender, education, income, and labor cost) and prior treatment group as dummy variables (4 years program, 2 years with technical assistance and 4 years with technical assistance). ESI values of previous periods have been included in both models to allow dynamic effects into the model. Table 1 shows the average value of the independent variables included in the analysis.

| Table A: Average values of independent variables included in the models | |
| --- | --- |
| *Variable* | *Average value* |
| Area (hectares) | 26.34 |
| Land sold (hectares) | 0.09 |
| Gender (1=male; 0=female) | 0.59 |
| Education max (number of years) | 6.08 |
| Income (1,000 COP) | 10,052.8 |
| Labor cost (1,000 COP) | 702.08 |
| 4 year PES without PES (1=yes; 0=no) | 0.08 |
| 4 year PES + technical assistance (1=yes; 0=no) | 0.26 |
| 2 year PES + technical assistance (1=yes; 0=no) | 0.20 |
| ESI 2003 per ha | 0.64 |
| ESI 2007 per ha | 1.19 |

Although our primary interest here is on changes in the post-PES period (2007-2011), we also run a model examining changes in the PES implementation period itself (2003-2007) with the same variables.

Models have been estimated using Ordinary Last Squares (OLS).

Table 2 shows the estimation results. The overall fit of the model is relatively low, especially for the PES period model (adjusted R^2^ equal to 0.31 and 0.59). This could indicate that other factors not included in the models might explain changes in ESI per hectare. However, error terms of both models follow a normal distribution and do not show other statistical problems in terms of considering spherical errors, like possibility of heteroscedasticity. They have been tested using Jarque-Bera test, for normality of error terms distribution, and White test, for heteroscedasticity.

The results for most explanatory variables are as expected, and similar to those of other studies on the adoption of agroforestry practices with small differences (Pagiola and Rios, 2013).

In Model 1 (period of PES implementation), PES programs have a positive and highly significant impact on ESI changes, as had already been shown by Pagiola and Rios [25]. Land and land sold have a negative impact on ES, though they are not very significant, while education has a positive and significant impact, and income has a positive impact, but again, they are not significant.

Variables from Model 2 (post-PES period) have similar signs (showing stability) but lower significance. Having previously participated in PES does not have a significant impact on changes in ES in the post-PES period. Most other variables also lose significance. These results are not surprising, given the very low level of variation in the dependent variable. Gender and labor costs are the only two variables that appear significant in this period. This could indicate that PES programs lose intensity over the time, but still have long-term effects, although the results are inconclusive. Moreover, other non-included variables could gain importance and still improve significance of programs.

| Table B. Estimation results | | | |
| --- | --- | --- | --- |
|  | | *Model 1* | *Model 2: Post-PES* |
| *Independent variable:* | | *ESI per ha 2007* | *ESI per ha 2011* |
| Ln [Area (hectares)] | | -0.019  (0.045) | 0.017  (0.021) |
| Land sold (hectares) | | -0.232  (0.158) | -0.051  (0.077) |
| Gender (1=man; 0=woman) | | -0.223  (0.168) | -0.186  (0.077)** |
| Education max (nº years) | | 0.035  (0.014)** | -0.004  (0.007) |
| Income (1,000 COP) | | 1.50E-06  (2.80E-06) | 1.57E-06  (1.35E-06) |
| Labor cost (COP) | | -4.12E-08  (3.78E-08) | -4.94E-08  (1.84E-08)** |
| 4 year PES (1=yes; 0=no) | | 0.308  (0.186)* | -0.041  (0.091) |
| 2 year PES +technical assistance (1=yes; 0=no) | | 0.343  (0.121)** | 0.052  (0.062) |
| 4 year PES +technical assistance (1=yes; 0=no) | | 0.549  (0.116)** | 0.026  (0.063) |
| Ln (ESI 2003 per ha) | | 0.419  (0.164)** | - |
| Ln (ESI 2007 per ha) | | - | 0.419  (0.053)** |
|  | |  |  |
| Period | | 2003-2007 | 2007-2011 |
| Adjusted R^2^ | | 0.31 | 0.59 |
| Number of observations | | 85 | 85 |
| *Notes*: | Standard errors in parentheses.  *, ** indicate coefficient estimate is significantly different from zero at 90% or 95% confidence level. | | |
